# Modeling of High Nanoparticle Exposure in an Indoor Industrial Scenario with a One-Box Model

**DOI:** 10.3390/ijerph16101695

**Published:** 2019-05-14

**Authors:** Carla Ribalta, Antti J. Koivisto, Apostolos Salmatonidis, Ana López-Lilao, Eliseo Monfort, Mar Viana

**Affiliations:** 1 Institute of Environmental Assessment and Water Research (IDÆA-CSIC), C/ Jordi Girona 18, 08034 Barcelona, Spain; apostolos.salmatonidis@idaea.csic.es (A.S.); mar.viana@idaea.csic.es (M.V.); 2 Chemistry faculty, University of Barcelona, C/ de Martí i Franquès, 1–11, 08028 Barcelona, Spain; 3 Institute for Atmospheric and Earth System Research (INAR), University of Helsinki, PL 64, FI-00014 Helsinki, Finland; joonas.koivisto@helsinki.fi; 4 Air Pollution Management, Willemoesgade 16, st tv, Copenhagen DK-2100, Denmark; 5 Institute of Ceramic Technology (ITC)- AICE - Universitat Jaume I, Campus Universitario Riu Sec, Av. Vicent Sos Baynat s/n, 12006 Castellón, Spain; ana.lopez@itc.uji.es (A.L.-L.); eliseo.monfort@itc.uji.es (E.M.)

**Keywords:** prediction, emission rates, air exchange rate, ultrafine particles, unintentional nanoparticles, incidental nanoparticles, plasma spraying, worker exposure, particle mass concentration

## Abstract

Mass balance models have proved to be effective tools for exposure prediction in occupational settings. However, they are still not extensively tested in real-world scenarios, or for particle number concentrations. An industrial scenario characterized by high emissions of unintentionally-generated nanoparticles (NP) was selected to assess the performance of a one-box model. Worker exposure to NPs due to thermal spraying was monitored, and two methods were used to calculate emission rates: the convolution theorem, and the cyclic steady state equation. Monitored concentrations ranged between 4.2 × 10^4^–2.5 × 10^5^ cm^−3^. Estimated emission rates were comparable with both methods: 1.4 × 10^11^–1.2 × 10^13^ min^−1^ (convolution) and 1.3 × 10^12^–1.4 × 10^13^ min^−1^ (cyclic steady state). Modeled concentrations were 1.4-6 × 10^4^ cm^−3^ (convolution) and 1.7–7.1 × 10^4^ cm^−3^ (cyclic steady state). Results indicated a clear underestimation of measured particle concentrations, with ratios modeled/measured between 0.2–0.7. While both model parametrizations provided similar results on average, using convolution emission rates improved performance on a case-by-case basis. Thus, using cyclic steady state emission rates would be advisable for preliminary risk assessment, while for more precise results, the convolution theorem would be a better option. Results show that one-box models may be useful tools for preliminary risk assessment in occupational settings when room air is well mixed.

## 1. Introduction

Thermal spraying is applied at an industrial scale to produce thermally- and mechanically-resistant coatings. A feedstock material (metal, alloy or ceramic) is projected at high temperature and velocity onto the surface to be coated. Protective coatings are widely used in the ceramic, automotive, naval, aeronautic and metallurgy industries to prevent corrosion and wear, as well as to restore different types of damaged surfaces [1,2,3,4,5]. From a risk assessment perspective, thermal spraying is known to generate unintentional nanoparticle (NP) emissions (with diameters < 100 nm; 10^6^ cm^−3^) at pilot-plant and industrial scales [6,7]. High particle mass concentrations have also been reported [8,9]. Similar NP emission and formation mechanisms have been identified in a large variety of industrial processes [10,11,12,13,14,15,16,17].

Identifying and modeling exposure to this kind of NPs is relevant due to their potential health impacts. Exposure to particulate matter (PM) may cause respiratory and cardiovascular diseases [18], where respirable particles (those which can penetrate into non-ciliated airways, EN 481, 1995) are considered as a harmful component for human health [18,19,20,21]. Oberdörster, (2001) [22] indicated the ability of finer size fractions (< 100 nm; NP) to penetrate deeper in the respiratory tract. In vivo studies have revealed that NPs can cross the alveolar barrier and cause pulmonary inflammation, which turns into cardiovascular risk [23] in much higher numbers than coarser particles, as they can reach deeper areas in the respiratory tract and therefore have a longer retention time [21]. 

Thus, unintentional NP release in workplaces is a key environmental health and safety issue which requires further research to understand the determinants of personal exposure. Mass-balance models are useful tools for this purpose, as they allow understanding of critical factors affecting exposure and could lead to efficient risk mitigation strategies [24]. Theoretical modeling aspects have been extensively discussed [25,26,27,28,29]. Indoor exposure assessment is frequently performed using one- and two-box models, which have been shown to predict exposure levels with relative accuracy when adequately parametrized in several environments (e.g., indoor concentrations of volatile compounds; PM concentrations in controlled and industrial scenarios [30,31]).

Key challenges for model application are source characterization, local controls and air mixing rates [30,32,33,34,35,36]. In addition, there is a need to test model performance under real-world conditions. Because of the large variety of indoor micro-environments and emission sources, libraries compiling model parameters would be highly useful for modeling studies [33,37]. Source characterization requires dedicated attention, as it is the main determinant of exposure. In the case of primary particle emissions, tools such as the dustiness index are available to estimate emission rates. However, as in the case of understanding exposure determinants in thermal spraying [6,7], emission rates must be quantified from measured concentrations using methods such as the convolution theorem or mathematical mass balance [27,38]. The use of estimated emission rates from actual monitored concentrations has been seen to provide good modeling results in laboratory studies [35,36,39]. However, literature related to emission rates of unintentional NP at industrial scale is limited [15,40]. As a result, emission rates for industrial sources of unintentional NP release and its use for real-world scenario modeling is highly valuable in view of the increasing use of prediction models and web-based risk assessment tools.

In this context, the aims of the present study are (1) to quantify NP emission rates for two different industrials scale thermal spraying processes; and (2) to test the performance of a one-box model in a real-world setting where high NP concentrations were monitored. 

## 2. Materials and Methods

### 2.1. Work environment and Process

**Figure 1:**
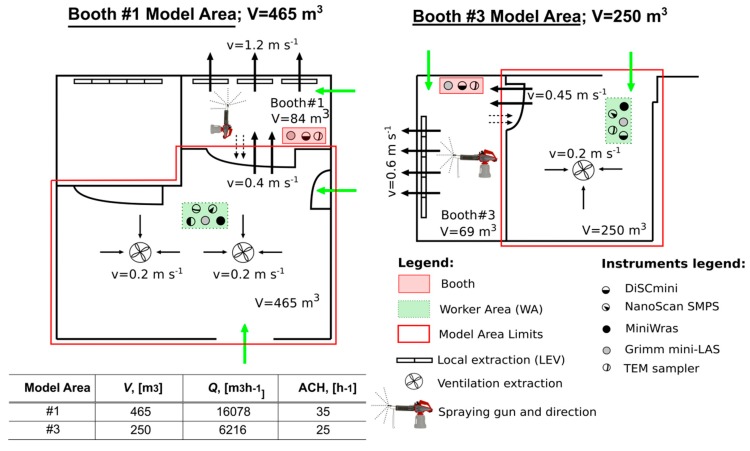
Modeled areas limit, volumes (m^3^), ventilation air speeds (m s^−1^), and description of instruments deployed and their location. Green and black arrows indicate incoming air flows and ventilation extraction flows, respectively. Dashed arrow indicates air flow from booth to WA. The table shows parameterization of the one-box model: V (m^3^) is volume used for modeling, Q (m^3^ h^−1^) is ventilation air volume flow through the WA, ACH (h^−1^) is the air changes per hour calculated from measured air speeds and used for modeling.

NP monitoring was carried out during thermal spraying of ceramic coatings in an industrial workshop (T.M. Comas, Blanes, Barcelona, Spain). Two types of spraying were applied, 1) High Velocity Oxy-Fuel coating spraying (HVOF), using low temperatures (< 3000 ºC) and high velocities; and 2) Atmospheric Plasma Spraying (APS), using high temperatures (up to 14000 ºC) [1,7,41]. Measurements were conducted over a 4-day period in April 2016. The workshop has three thermal spraying booths in an area of approximately 240 m^2^ (14 m wide and 17 m in length) (Figure 1). Here, only data from booth #1 (APS) and #3 (HVOF) will be considered for the modeling approach (Figure 1 and Table S1, Supplementary material) given that booth #2 was not operated continuously. The central area of the workshop (outside the booths), from now on called the worker area (WA) is equipped with a general ventilation extraction system that consisted of three extractors with 11800 m^3^ h^−1^ flow each (value provided by the company, not measured). Booth #3 and #1 were additionally equipped with a localized extraction ventilation system (LEV), but only Booth #3’s LEV air flow rate was provided by the company (6500 m^3^ h^−1^). Booth #3’s activity periods had a short duration and high frequency (5–10 min, 7–9 repetitions/half day) whereas in booth #1, periods had a longer duration and lower frequency (20–30 min, 2–4 repetitions/half day). The two booths were not operated at the same time. However, certain activities such as the cooling and sanding of pieces were occasionally carried out in parallel. More details on the campaign can be found in Salmatonidis et al. (2019) [7]. In booth #1 the door was usually closed while spraying although not completely, thus allowing air mixing between the booth and the WA. In booth #3, during the activity the worker had to enter and leave the booth more frequently due to technical needs of the process and increased the air mixing between the booth and the WA. 

### 2.2. Feedstock Material 

In booth #1 (days 1 and 2), two types of commercial products (feedstock) were sprayed: Amdry 6228 (Oerlikon Metco, Pfäffikon, Switzerland) and ANVAL 50/50 (Anval) (Table S1). The Amdry 6228 formula is Al_2_O_3_ 13TiO_2_, and it consists of alumina 84% (CAS: 1344-28-1), 14% titania (CAS: 13463-67-7), and organic binder silicon dioxide (CAS: 7631-86-9). The mean aggregate size is 36.0 μm [7] and particles are considered angular/blocky. Amdry is classified as dangerous according to Directive 1999/45/EC [42] and its amendments. The ANVAL 50/50 formulation consists of 50% chromium (CAS: 7440-47-3) and 50% nickel (CAS: 7440-02-0). The powder’s mean aggregate size is 76.5 μm [7]. The fact that instruments were intercompared using a different type of aerosol is acknowledged as a limitation of this work.

The feedstock sprayed in booth #3 (days 3 and 4) was a WOKA 3702–1 powder (Oerlikon Metco, Pfäffikon, Switzerland) (Table S1). Its formula is WC 20Cr_3_C_2_ 7Ni, and it consists of 69.5% tungsten carbide (CAS: 12070–12–1), 14.5% trichromium dicarbide (CAS: 12012-35-0), 9% chromium (CAS: 7440-47-3) and 7% nickel (7440-02-0). An unspecified portion of metallic chromium and nickel may be converted during the thermal spray process to hexavalent chromium and nickel compounds, respectively, which are classified as IARC group 1 carcinogen (Safety data sheet, Supplementary material). The mean aggregate size is 34.3 μm [7] and particles are considered mainly spheroidal with an apparent density between 3.8–4.9 g cm^−3^. WOKA 3702–1 is classified as hazardous according to Regulation (EC) 1272/2008 [43] and it is suspected to cause cancer.

### 2.3. Online Measurements and Airborne Particle Collection

Mean particle diameter, and particle number and mass concentrations were monitored inside the booths and in the central WA (Figure 1). Additionally, particle size distribution was monitored in the WA. 

The monitoring instruments deployed were:

- A miniature diffusion size classifier (DiSCmini Matter Aerosol, Testo; sample flow rate 1 l min^−1^) to measure particle number concentration, mean particle size and alveolar lung deposition surface area (LDSA) in a range of 10 to 700 nm, with a 1-minute time resolution.
- A Mini Laser Aerosol Spectrometer (Grimm, Mini-LAS 11R; sample flow rate 1.2 L min^−1^) to measure particle mass concentration from 0.25 to 32 µm in 31 channels, with a 1-minute time resolution.

Inside the spraying booths, instruments were located near the cabin extraction system, and at 1.5 m from the plasma spray nozzle. 

The WA instruments were located outside the booths, at approximately 4 m from the door (Figure 1). The instruments were located between 0.5 and 1.5 m above ground. Concentrations were monitored by using a DiSCmini and a Grimm Mini-LAS (as described above), as well as:

- An electrical mobility spectrometer (NanoScan, SMPS TSI Model 3910; sample flow rate 0.7 L min^−1^) to measure particle number concentration and size distribution in 13 channels from 10 to 420 nm, with a 1-minute time resolution.
- A Mini Wide Range Aerosol Spectrometer (Mini-WRAS 1371; sample flow rate 1.2 L min^−1^) to measure particle mass concentration, number concentration and size distribution from 10 nm to 35 µm in 41 channels, with a 1-minute time resolution.

Particle metrics were monitored each day between 10:00 and 17:00. During the lunch breaks, from 12:45 to 13:45, processes were switched off and this period was considered to be representative of background (BG) concentrations.

Instrument intercomparison was carried out prior to the industrial measurements at an air quality monitoring station in Barcelona, Spain, using ambient air aerosols [7]. The fact that instruments were intercompared using a different type of aerosol is acknowledged as a limitation of this work.

Increases in particle number and respirable mass concentrations during spraying were considered statistically significant when the following approach, described by Asbach et al. (2012) [44] and Kaminski et al. (2015) [45], was fulfilled:

Mean concentration during spraying > BG ± 3∙(σBG),(1)

where BG is the mean temporal background concentration and σBG is the standard deviation of the BG concentration. 

Finally, in order to complement the air flow data provided by the company, air speeds at booths LEV, central ventilation extraction, and inside the plant were experimentally measured (shown in Figure 1) with an anemometer (VelociCalc ^®^ thermal anemometer, TSI Model 9545; TSI Inc., Shoreview, MN, USA; range 0–30 m s^−1^; accuracy ±3% or ±0.015 m s^−1^; resolution 0.01 m s^−1^).

### 2.4. Exposure Modeling

#### 2.4.1. Air changes per hour (ACH)

The number of air changes per hour (ACH) in the WA was calculated by using the measured air speeds in the central ventilation extraction and also next to the booth doors, as:

ACH = Q/V = (m^3^ h^−1^)/m^3^, (2)

where Q (m^3^ h^−1^) is the total flow rate and V (m^3^) is the total room volume.

#### 2.4.2. Particle Emission Rates

Particle emissions were monitored in close proximity to the source (the spraying nozzle), with the logistical limitations characteristic of operational industrial scenarios, where measurements should be as least invasive as possible. Despite the air extraction systems in place, particle number concentrations measured inside the booths largely exceeded the instrument (DiSCmini) monitoring range (4 × 10^6^ cm^−3^; Figure 1 and Figure S1 in Supplementary materials). As a result, particle emissions rates could not be calculated based on data collected inside the booths. To overcome this limitation, emission rates were calculated based on the data collected in the WA, i.e., how much particles are leaking from the booth to the WA (booth is the source). This was based on the assumption that air exchange between both areas was frequent: the booth doors were sometimes wide open (when the operator had to access the inside of the booth), and when they were closed they were not fully airtight, and small holes through which cables were inserted were visible along the booth walls. As a result, particle emissions in the booths impacted concentrations in the WA significantly [7]. Similar results have been reported by other authors [46], indicating impacts on particle number concentrations up to 6 m away from the source. 

In the present work, particle emission rates were estimated from concentrations measured in the WA, and thus do not strictly correspond to actual emission rates but are rather considered as transported emission rates from the booths to the WA. These transported emissions were used as input to model particle concentrations in the WA. For the sake of clarity and in the present work, transported emission rates from the booths to the WA, measured in the WA, will be referred to as emission rates.

Emission rates were calculated by using two different approaches:

##### Convolution Theorem

Assuming that NP concentrations are fully mixed, the WA particle concentrations can be described with a mass balance of aerosol particles in a single compartment [28]:

(dN(t))/dt = λN_BG_ (t) + (S_N_ (t))/V–λN(t), (3)

where N (cm^−3^) is the WA particle concentration, λ (min^−1^) is the WA total particle loss rate including particle removal processes like ventilation and deposition, N_BG_ (cm^−3^) is the background particle concentration coming from outdoors and surrounding compartments, S_N_ (min^−1^) is the particle emission rate of the source, and V (m^3^) is the volume of the WA room.

The one-box model [28] assumes that 1) particles are fully mixed at all times; 2) particles are created by the source and through infiltration; and 3) there are no other particle losses than mechanical ventilation. Particle losses by sedimentation may be considered negligible because of the high air exchange ratio (ACH) measured (25–35 h^−1^, Figure 1).

When background particle emissions and indoor sources are negligible (i.e., S_tot_ (t) ≈ 0 min^−1^), e.g., during the lunch break, the particle number concentration decay curve may be described as follows:

N(t) = N_t=0_ ∙ e^-γ∙t^, (4)

The calculated ACH varied from 25 to 35 h^−1^, which is much higher than particle deposition rates, which are typically below 4 h^−1^ in indoor environments for particles within the measurement range in this work (10 nm to 35 µm; [47]). Thus, the only particle loss considered is through ventilation, and here it was assumed that λ ≈ ACH.

According to the convolution theorem, the particle number concentration during the activity is a convolution of the particle sources and particle losses as follows [38]:

S_tot_(t) ∙ N(t) = V_0_ʃ^t^(QN_BG_ (t) + ε_C_ S_N_ (t)) ∙ N(t-τ)dτ, (5)

where Q (m^3^ h^−1^) is the air flow and ε_C_ (-) is the protection factor of the booth.

The particle emission term can be solved with a numerical deconvolution as:

S_tot_ (t) = V∙ (N(t) - N(t-∆t) ∙ e^-γ∙∆t^)/∆t, (6)

In this work, particle emission rates were calculated for the pre-activity period when S_N_ (t) = 0 min^−1^ to estimate background particle generation rate S_BG_ (t) = QN_BG_ (t) (corresponding mainly to infiltration from outdoor air). Because the booth protection factor ε_C_ was not known, the plasma spray emission rate term is a combination of ε_C_ S_N_ where background particle emission rate S_BG_ is subtracted.

##### Steady State Equation for Cyclic Processes

In addition, emission rates during the activity were also calculated using particle number concentrations (cm^−3^) monitored in the WA with the NanoScan instrument, by applying the modified steady state equation for cyclic processes [7]:

S_N_ = Ĉ∙V/t_ESD_, (7)

where Ĉ (cm^−3^) is the mean concentration of particles (as number concentration) during spraying, V (cm^3^) is the volume of the room, and t_ESD_ (min) is the spraying duration. Assumptions were that particle concentrations before the activity were lower than during the activity, particle removal processes were negligible, and that particle concentrations were fully mixed.

#### 2.4.3. One-box Mass Balance Model

As particle concentrations monitored inside the booths exceeded the instrument’s measurement range, applying a two-box model to estimate particle concentrations inside and outside the booths was not possible. Therefore, a one-box model was applied to calculated particle number concentrations. With the emission rates calculated in the WA, the one-box model was applied to the WA volume considering the influence of two types of ventilation systems: the central one in the WA, and the ventilation driving the air flows from the WA to the booth due to the booth’s LEV (Figure 1). Booth air extraction systems were compensated with air coming from the WA (through the door and through small holes for cables) and from outdoors (through a direct pipeline connecting with outdoor air). Background particle concentrations were included in the model with the incoming air, as this was seen to improve mass-balance model performance and accuracy [33].

## 3. Results and Discussion

### 3.1. Exposure Concentrations

**Figure 2:**
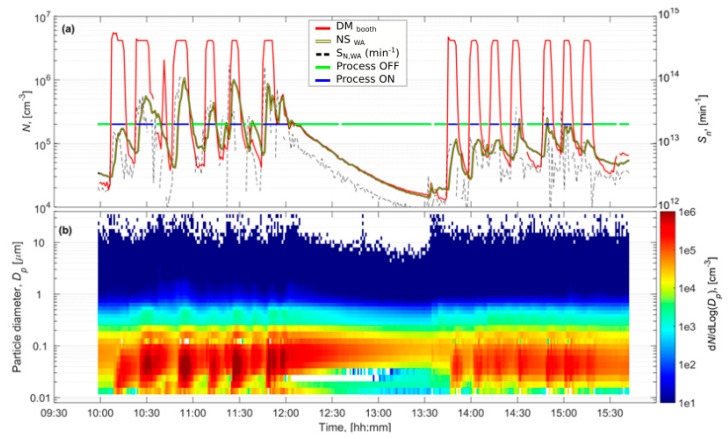
Booth #3, Day 3 **(a)** shows the particle number concentrations measured inside the booth by DiSCmini (DM), from worker area by NanoScan (WA) and particle emission rates solved by convolution from NanoScan WA concentrations. Blue line shows when the DM concentration was >10^5^ cm^−3^ indicating that the plasma spray was ON and green line when the DM concentration was <10^5^ cm^−3^ indicating that the plasma spray was OFF. Figure **(b)** shows the particle size distributions measured by the NanoScan in the WA.

Particle number concentrations (Table 1) were monitored during thermal spraying in booth #1 (APS, days 1 and 2) and #3 (HVOF, days 3 and 4). Respirable particle mass concentrations varied from 130 to 709 µg m^3^ inside the booths, and 93 to 172 µg m^3^ in the WA (Table S2, Supplementary material), which is below the occupational exposure limits (OELs) given in the material safety data sheet (MSDS). A clear impact on particle number concentrations was observed inside the booths and in the WA, once the spraying activity started (Figure 2 and Figures S2 and S3, Supplementary material). Additionally, chemical analyses of the airborne particles indicated that they had the same composition as the feedstock material being sprayed (more details in Salmatonidis et al., 2019 [7]). Thus, it was concluded that the spraying activities generated high concentrations of unintentionally emitted NPs. Chemical analyses of the airborne particles indicated that their composition in terms of mass was that of the raw feedstock material, and that chromium was present in the form of chromium carbide and nickel in its free form (more details in Salmatonidis et al., 2019 [7]). 

The particle number concentrations, measured with the DiSCmini inside the booths during spraying, ranged between 8.5 × 10^5^ and 3.5 × 10^6^ cm^−3^ in booth #1, and between 1.0 × 10^6^ and 1.9 × 10^6^ cm^−3^ in booth #3 (Table 1), exceeding instruments detection limit (Figure 2 and Figures S1, S2 and S3, Supplementary material). The operators wore personal protective equipment (FPP3, masks in booth #3, and welding helmet with respirator in booth #1) when they entered the booths but in the WA, the operators only wore the masks intermittently. NP concentrations were approximately one order of magnitude lower than inside the booths (6.4 × 10^3^-9.6 × 10^4^ cm^−3^ and 1.1-3.5 × 10^5^ cm^−3^ for booth #1 and #3, respectively). However, the impact of booth #1 emissions on WA concentrations (especially during day 1) was lower than that of booth #3 emissions (Table 1). Particle concentrations recorded in the WA by the NanoScan monitor were similar to the ones measured by the DiSCmini: 4.2-7.8 × 10^4^ cm^−3^ and 9.0 × 10^4^-2.5 × 10^5^ cm^−3^, for booth #1 and #3, respectively. Due to the similarity between both datasets, and the fact that the NanoScan provided additional information on particle size distributions, the latter dataset was used as an input for the one-box model.

In booth #3 the worker had to enter the booth between repetitions to exchange the coated surface with a new one (to be coated). As the spraying periods were short (5–10 min), the door of the booth was kept open between repetitions during Day 2/Morning, whereas during Day 2/Afternoon the door was alternatively open and closed. This explains the significantly lower concentrations measured in the WA during Day 2/Afternoon compared to during the Morning (Figure 2).

In all cases (inside the booths, and in the WA except on Day 1), particle concentrations exceeded the 40,000 cm^−3^ threshold considered the nano-reference value used in the precautionary approach [48], and they were statistically significantly higher than background concentrations [44], Eq. 1).

### 3.2. Air Exchange Quantification

Air speeds were measured at the central ventilation system and at the doors of the booths, during activity periods, and were used to quantify the total air flows in the WA during spraying. The total air changes per hour (ACH, h^−1^) were then estimated by considering the volume of each of the modeled areas (to reproduce emissions from booths #1 and #3, Figure 1). The total flows (Q) for model area #1 and #3 were 16078 and 6216 m^3^ h^−1^, respectively with ACH values of 35 and 25 h^−1^. These values are quite high although in line with reported values in industrial environments, which typically range between 0.3-30 h^−1^ [49,50]. Additionally, air speeds were measured at the booth doors when they were open, with ACH ranging between 49–89 h^−1^ (Table S3). The latter ACH are not representative of actual working conditions, given that doors were mostly closed when the spraying guns were operating. Overall, the ACH measured indicate a high variability of the air exchange rates in the workplace, which would influence the modeling results as the ACH is, together with the emission rate, a key input parameter for the model.

### 3.3. Particle Emission Rates

Particle emission rates were estimated based on WA concentrations, using convolution and cyclic steady state equations (Table 2) in order to compare the results from both approaches. Results showed that emission rates from booth #1 ranged between 1.4 × 10^11^–3.4 × 10^12^ min^−1^ using the convolution theorem, and between 1.3 × 10^12^–3.0 × 10^12^ min^−1^ using the cyclic steady state equation. Both approaches provided similar outputs for booth #3, ranging between 7.9 × 10^12^–1.2 × 10^13^ min^−1^, with the convolution theorem and between 7.9 × 10^12^–1.4 × 10^13^ with the cyclic steady state. Emission rates were higher from booth #3 than from booth #1, which is consistent with the different operational procedures (HVOF vs. APS; [7]).

In general, both methods provided relatively similar results despite the differences in calculations, thus supporting the robustness of the emission rates calculated. The cyclic steady state generally provided higher emission rates than the convolution theorem (from 1.8 up to 10 times higher), with the exception of Day 3 in booth #3, for which the convolution theorem provided a higher rate (by a factor of 4–16; Table 2). When using the cyclic steady state, differences between days in the same booth were smaller than when using the convolution theorem, suggesting that the convolution theorem is more case-sensitive and will, therefore, probably provide more precise modeling results. In Koivisto et al. (2018) [15], where particle number emission rates were quantified during electrostatic spray deposition of TiO2 by using the same approaches as in the present study, emission rates near the source were 2.2 × 10^12^ min^−1^ (convolution theorem) and 1.1 × 10^12^ min^−1^ (cyclic steady state). As in the present work, emission rates calculated with the two methods were mostly comparable despite differences in the calculation methods.

The emission rates quantified for the thermal spraying activities were comparable to other emission sources in the literature, e.g., dip coating (4.2 × 10^11^ min^−1^ and 6.6 × 10^11^ min^−1^, [40], laser printing (maximum rates between 2.4 × 10^9^ and 1.0 × 10^13^; [38,51,52]), in houses due to Tabaco (0.84-3.76 × 10^11^ min^−1^) and cooking (1-8 × 10^11^ min^−1^) [53,54,55], or NP production in academic laboratories (1.3 × 10^11^–1.2 × 10^12^ min^−1^; [56]), among others.

### 3.4. One-Box Model Performance

Worker area (WA) particle number concentrations were modeled by applying a one-box mass balance model [28]. The two types of emission rates calculated (using convolution theorem and cyclic steady state approach) were used as input, with the aim to test model performance under a real-world industrial scenario. The one-box model was applied to the booth #1 and #3 model areas, to predict particle number concentrations in the WA during thermal spraying from each of the booths. The model was tested for two days (including one morning and one afternoon shift, each) in each model area (#1 and #3).

**Figure 3:**
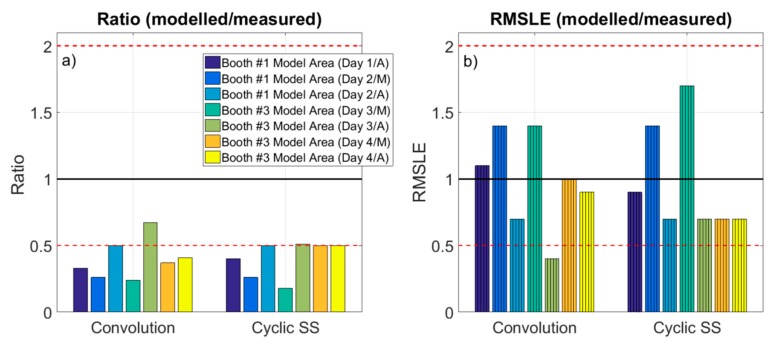
Ratio modeled/measured concentrations **(a)**, and RMSLE (modeled/measured) **(b)**. Ratio 0.5 and 2 are marked as reference (dashed line). Cyclic SS: cyclic steady state equation.

Modeled particle number concentrations in the WA based on the NanoScan monitored concentrations ranged between 1.4–2.4 × 10^4^ (convolution theorem) and 1.7–2.4 × 10^4^ (cyclic steady state) while spraying in booth #1, and between 5.3–6.0 × 10^4^ (convolution theorem) and 4.5–7.1 × 10^4^ (cyclic steady state) while spraying in booth #3 (Table 2). When comparing these concentrations with actual measured NanoScan ones, on a case by case basis, results indicate that in all cases the model underestimated measured concentrations irrespective of the method used to calculate emission rates: ratios modeled/measured concentrations were < 0.5 for all of the study cases (with one exception, Figure 3a). When comparing modeled concentrations to measured DiSCmini concentrations in the WA, ratios modeled/measured were consistent with those obtained with NanoScan (<0.55, except for the booth #1, day 1), also underestimating measured concentrations (Table 2 and Figure 3a). In general, higher underestimations and variability of the measured concentrations were obtained when using the DiSCmini dataset for model validation. These differences may be attributed to the fact that both instruments (NanoScan and DiSCmini) are not directly comparable as DiSCmini (corona charging principle) monitors particle diameters between 10–700 nm while NanoScan (single particle counting) monitors 20–420 nm (Fonseca et al., 2016). It should be noted that concentrations in terms of particle number are significantly higher than in terms of mass, which is a metric more frequently used with mass balance models, and both metrics are known to represent different particle properties (e.g., size, density, etc.) and therefore have different behaviors [56].

Because of this limitation, the assessment of the root mean squared logarithmic error (RMSLE) is proposed to not over-penalize differences between predicted and actual values, when concentrations are high [36]. This is because the RMSLE mainly takes into account the ratio of change, rather than the actual concentrations. Figure 3b shows that the RMSLE (modeled/NanoScan measured) ranged between 0.4–1.4 when using convolution emission rates and 0.7–1.7 when using cyclic steady state. The same analysis using the DiSCmini dataset resulted in RMSLE (modeled/DiSCmini measured) between 0.6–1.8 when using convolution emission rates and 0.9–2.1 when using cyclic steady state (data not shown). These ratios were compared to the benchmarks proposed by Jayjock et al. (2011) [30], which representative of modeling outputs reviewed in literature. Thus, when considering the RMSLE, only one case was outside the Jayjock et al. (2011) benchmark (0.5–2): booth #3 (Day3/A) with the convolution theorem. This would mean that, according to the ranges suggested by Jayjock et al. (2011), the performance of the one-box model for the industrial scenario assessed in this work would be considered as comparable. In the literature, several authors have reported underestimations by the one-box model [31,35,36,57,58] as it assumes homogeneous concentrations through all the room. This is consistent with our results, even though the high airflows measured in the industrial scenario allowed us to assume adequate mixing, on average. However, it is possible that full mixing was not achieved at all times and during all repetitions, and this should be considered as one of the limitations of the present work.

Model underestimations could also be related to inaccurate ACH calculation or to the underestimation of emission rates including booth protection factor. Emission rates are known to be the most critical exposure determinant, and thus should be calculated based on experimental data collected as close (physically) to the source as possible with known dilution and mixing. Otherwise, large differences may be detected depending on the location and position of the monitoring instrumentation [36]. These authors demonstrated that emission rates can be corrected by using an adjusting coefficient to compensate for unknown flow distributions. The results presented in this work, which are based on emission rates calculated from data collected at a distance from the source (>3m), support the need for a correction coefficient to improve the emission rates calculated. Unfortunately, knowledge of air mixing is a frequent issue encountered when measurements are carried out in real-world industrial scenarios.

**Figure 4:**
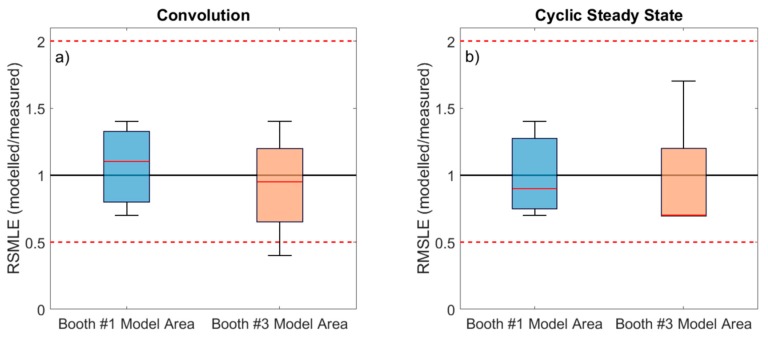
Vertical box plot for RMSLE modeled/measured concentrations for booth#1 and booth#3 model areas using convolution SN **(a)** and Cyclic steady state SN **(b)**. Ratio 0.5 and 2 are marked as reference (dashed line). The boundary of the box closest to zero indicates the 25th percentile and the farthest from zero the 75th percentile. The solid red line within the box indicates the median value. Error bars above and below indicate the 10th and 90th percentiles.

The results were also analyzed in terms of average RMLSE across all cases (Days 1 to 4, morning and afternoon shifts), for the booth #1 and #3 scenarios (Figure 4). Results show that when emission rates were calculated using the convolution theorem, model predictions were more closely aligned with RMLSE = 1 than when the cyclic steady state equation was used, and the ratios (especially for booth #3) presented lower variability. The results obtained when applying the convolution theorem were more case-sensitive, whereas applying the cyclic steady state required lower computational efforts. Overall, differences obtained with both parametrizations were not large. Therefore, for preliminary risk assessment and based on the data from this industrial scenario, particle emission rates calculated with the cyclic steady state equation may be sufficient, whereas for a more case-specific analysis the convolution theorem may be a better approach for emission rate calculation.

Finally, model performance was also evaluated with regard to the type of particles emitted, which were different in the two scenarios given that different thermal spraying techniques were used (APS in booth #1, and HVOF in booth #3). In short, particles emitted in booth #1 were mostly spherical and resulting from melting of the ceramic powder sprayed, while particles emitted in booth #3 showed more irregular morphologies resulting from mechanical impaction of the powder on the surface to be coated [7]. Modeling results for both scenarios showed only slight differences (Figure 4), which did not seem to be linked to the spraying techniques (APS and HVOF) but rather to the layout and configuration of the study area (Figure 1). In the booth #1 model area the door was always kept in a similar position (partially closed). Conversely, in the booth #3 model area, due to requirements of the spraying process the worker had to enter and exit the booth frequently, which means that the door configuration and the number of times that it was open and closed was different in every shift. The door configuration not only impacts particle transport, but also air flows (ACH). The impact of this can be clearly observed in Day 3 (Figure 2), where markedly higher concentrations were recorded in the WA during the morning (when the door was open) than in the afternoon (door closed), while emissions at the source remained at relatively constant levels. Given that particle emission rates were calculated for full days, this intra-daily variability is considered a source of the higher variability in modeled/measured ratios (Figure 3) and RMLSE (Figure 4) observed for booth #3 (in comparison with booth #1). This result indicates that, to improve model performance, not only emission rates but also the ACH need to be calculated as precisely as possible, given that small variations can lead to significant under- or overestimations of particle concentrations and, thus, can limit the effectiveness of risk assessments and lead to impaired decision-making. 

An example of the variability of model outcomes is reported in Supplementary material (Table S3 and S4), using a different door configuration (door fully open). The estimated ACH values from measured air speeds when booths doors were open ranged between 49–89 h^−1^, which is approximately double than the ACH (25–35 h^−1^) estimated when booths doors were closed. Modeled/measured concentrations ratios using ACH 49–89 h^−1^ ranged between 0.1–0.4, indicating higher underestimations of measured concentrations than when using ACH 25–35 h^−1^ (when ratios ranged between 0.2–0.7, Table 2). As door configurations and worker layout were not always constant, especially in booth #3 where the time during which the door was open/closed was different in every repetition, estimated ACH values used for the modeling (25–35 h^−1^) may not be fully representative of actual ACH values for each specific repetition. In such cases, were ACH may be highly variable with time, a probabilistic approach or a sensitivity analysis as the one conducted in Ribalta et al., 2019 [33] would be highly useful.

According to the literature, mass balance models have been satisfactorily tested in real-world settings mostly in terms of particle mass [39,59,60,61,62] with ratios modeled/measured of 0.82–1.22 and 1.10 ± 2.32 during packing and pouring processes [32,33], and for vapors and sprays with ratios of 0.49–3.29 and 0.32–3.28 for chemical component emissions [30,63]. However, models have not been extensively tested for particle number concentrations, which is currently the most commonly-used metric for NP exposure, in real-work environments. In Jensen et al. [35,36], one-, two- and three-box models were tested for particle number concentrations from a brush generator in a controlled chamber experiment, with models being able to estimate concentrations within a RMSLE factor of 0.5–2 [36].

In the present work, ratios for modeled concentration were 0.2–0.7 and RMSLE 0.4–1.7, with the model clearly underestimating actual exposures. It may thus be concluded that the one-box model can provide a preliminary idea of the order of magnitude of unintentional NP concentrations, and that the accuracy of the results depends on the input parameters like ACH and particle emission rates. Therefore, the availability of a publicly-shared emission-rate library for modeling would be highly useful for preliminary risk assessments in absence of measured emission rates or particle concentrations. However, more precise modeling results require accurate quantification of the input parameters, implying that additional work in model parametrization is necessary. Model underestimations, even when ratios are within the 0.5–2 benchmark, are undesirable from a risk assessment point of view, where overestimating concentrations would be more precautionary.

## 4. Conclusions

An industrial scenario characterized by high concentrations of unintentionally-released NPs was selected to test the performance of a one-box mass balance model. Emission rates in terms of particle number concentration, one of the main inputs of the model, were estimated using two approaches: the convolution theorem and the cyclic steady state equation. The main conclusions extracted are:

- Nanoparticle emission rates from thermal spraying of ceramic coatings were from the booth to working area (WA) in the range 1.4 × 10^11^–1.4 × 10^13^ min^−1^. These emission rates are slightly higher or of a similar order of magnitude as those reported in the literature from sources such as industrial dip-coating, laser printing or even indoor cooking in homes.
- Both approaches for emission rate calculation provided comparable rates, which were slightly lower when the convolution theorem was used. The cyclic steady state approach required lower computational efforts.
- The one-box model underestimated concentrations measured in the WA irrespective of the method used for emission-rate calculation. The ratios modeled/measured concentrations were 0.2–0.7 (using the convolution theorem) and 0.2–0.5 (using the cyclic steady state equations). When correcting high concentrations by using the root mean squared logarithmic error (RMSLE), ratios were 0.4–1.4 (convolution) and 0.7–1.7 (cyclic steady state).
- Even though both model parametrizations showed similar performance on average, the use of emission rates calculated with the convolution theorem improved results on a case by case basis (results were more case-sensitive). Thus, emission rates calculated with the cyclic steady state approach would be advisable for preliminary risk assessment, while for more precise case-specific results, the convolution theorem would be the better option.
- An additional key input affecting model performance was seen to be the estimation of the air exchange per hour (ACH). This parameter was strongly impacted by the position of the door of the booth where emissions were generated, which should be taken into account carefully in modeling exercises.
- In sum, with adequate parametrization, one-box mass balance models may provide useful guidance regarding the order of magnitude of expected particle number concentrations in industrial scenarios, and thus be used as a preliminary risk assessment tools.

## Figures and Tables

**Table 1:** Measured particle number concentration during the thermal spraying activity. Statistically significant increases are marked in bold.

Day	Shift	Booth	Worker Area (WA)		Inactivity (BG)	
		DiSCmini N (cm^−3^)	DiSCmini N (cm^−3^)	NanoScan N (cm^−3^)	DiSCmini N (cm^−^^3^)	NanoScan N (cm^−^^3^)
Booth #1 Model Area (Day 1)	Afternoon	**3.5 × 10^6^**	6.4 × 10^3^	4.2 × 10^4^	1.2 × 10^4^	1.4 × 10^4^
Booth #1 Model Area (Day 2)	Morning	**1.1 × 10^6^**	**9.6 × 10^4^**	**7.8 × 10^4^**	1.2 × 10^4^	1.7 × 10^4^
	Afternoon	**8.5 × 10^5^**	**6.7 × 10^4^**	**4.9 × 10^4^**		
Booth #3 Model Area (Day 3)	Morning	**1.9 × 10^6^**	**3.5 × 10^5e^**	**2.5 × 10^5^**	2.0 × 10^4^	1.9 × 10^4^
	Afternoon	**1.7 × 10^6^**	**1.1 × 10^5^**	**9.0 × 10^4^**		
Booth #3 Model Area (Day 4)	Morning	**1.3 × 10^6^**	**1.9 × 10^5^**	**1.5 × 10^5^**	4.5 × 10^4^	3.7 × 10^4^
	Afternoon	**1.0 × 10^6^**	**1.6 × 10^5^**	**1.3 × 10^5^**		

**Table 2:** One-Box modeled concentrations using the convolution theorem and the cyclic steady state (Cyclic SS) approach to calculate emission rate (S_N_) from NanoScan data. Emission rates were calculated for each day considering all activity periods, and modelings were applied for morning (M) and afternoon (A) periods (shift) separately.

Model Area		Booth #1			Booth #3			
Day		Day 1	Day 2		Day 3		Day 4	
Shift		A	M	A	M	A	M	A
**WA NanoScan S_N_**	**Convolution**	1.4 × 10^11^	3.4 × 10^12^		1.2 × 10^13^		7.9 × 10^12^	
	**Cyclic SS**	1.3 × 10^12^	3.0 × 10^12^		7.9 × 10^12^		1.4 × 10^13^	
**Modeled concentrations (cm^−3^)**	**Convolution**	1.4 × 10^4^	2.0 × 10^4^	2.4 × 10^4^	5.9 × 10^4^	6.0 × 10^4^	5.6 × 10^4^	5.3 × 10^4^
	**Cyclic SS**	1.7 × 10^4^	2.0 × 10^4^	2.4 × 10^4^	4.5 × 10^4^	4.6 × 10^4^	7.1 × 10^4^	6.5 × 10^4^
**WA measured** **(cm^−3^)**	**NanoScan**	4.2 × 10^4^	7.8 × 10^4^	4.9 × 10^4^	2.5 × 10^5^	9.0 × 10^4^	1.5 × 10^5^	1.3 × 10^5^
	**DiSCmini**	6.4 × 10^3^	9.6 × 10^4^	6.7 × 10^4^	3.5 × 10^5^	1.1 × 10^5^	1.9 × 10^5^	1.6 × 10^5^
**Ratio** **(modeled/NanoScan measured)**	**Convolution**	0.33	0.26	0.49	0.24	0.67	0.37	0.41
	**Cyclic SS**	0.40	0.26	0.49	0.18	0.51	0.47	0.50
**Ratio** **(modeled/DiSCmini measured)**	**Convolution**	2.19*	0.21	0.36	0.17	0.55	0.29	0.33
	**Cyclic SS**	2.66*	0.21	0.36	0.13	0.42	0.37	0.41

* Unusually high ratios were obtained for Day 1, probably due to the low impact of particle emissions on WA concentrations (Figure S1) which led to an overestimation of low concentrations by the model.

## Supplementary Materials

The following are available online at https://www.mdpi.com/1660-4601/16/10/1695/s1, Figure S1: Booth #1, Day 1 a) shows particle number concentrations measured inside the booth by DiSCmini (DM), from worker area by NanoScan (WA) and particle emission rates solved by convolution from NanoScan WA concentrations, Figure S2: Booth #1, Day 2 a) shows particle number concentrations measured inside the booth by DiSCmini (DM), from worker area by NanoScan (WA) and particle emission rates solved by convolution from NanoScan WA concentrations, Figure S3: Booth #3, Day 4 a) shows particle number concentrations measured inside the booth by DiSCmini (DM), from worker area by NanoScan (WA) and particle emission rates solved by convolution from NanoScan WA concentrations, Table S1: Sampling day, booth, technic used (HVOF or APS), and feedstock materials summary, Table S2: Respirable mass concentration during the thermal spraying activity, Table S3: Parameterization of the single box model considering booth door open: V (m3) is volume used for modeling, Q (m3 h–1) is ventilation air volume flow through the Worker Area and ACH (h–1) is the air changes per hour calculated from measured air speeds, Table S4: One Box modeled concentrations considering booth door open, and using the convolution theorem and the cyclic steady state (Cyclic SS) approach to calculate emission rate (SN) from NanoScan data.
