# Supplementary material for: Modeling of High Nanoparticle Exposure in an Indoor Industrial Scenario with a One-Box Model
Source: Int J Environ Res Public Health. 2019 May 14;16(10):1695. doi: 10.3390/ijerph16101695 (PMC6572703; doi:10.3390/ijerph16101695)
Supplement: Supplementary file 1 [file ijerph-16-01695-s001.pdf]

Supplementary materials

Modeling of High Nanoparticle Exposure in an Indoor Industrial Scenario with a One-Box Model

Carla Ribalta [1,2,*], Antti Joonas Koivisto[3,4], Apostolos Salmatonidis[1,2], Ana López-Lilao[5], Eliseo Monfort[5], Mar Viana[1]

1 Institute of Environmental Assessment and Water Research (IDÆA-CSIC), C/ Jordi Girona 18, 08034 Barcelona, Spain
2 University of Barcelona, Chemistry faculty, C/ de Martí i Franquès, 1-11, 08028 Barcelona, Spain
3 University of Helsinki, Institute for Atmospheric and Earth System Research (INAR), PL 64, FI-00014 Helsinki, Finland
4 Air Pollution Management, Willemoesgade 16, st tv, Copenhagen DK-2100, Denmark
5 Institute of Ceramic Technology (ITC)- AICE - Universitat Jaume I, Campus Universitario Riu Sec, Av. Vicent Sos Baynat s/n, 12006 Castellón, Spain
* Correspondence: carla.ribalta@idaea.csic.es

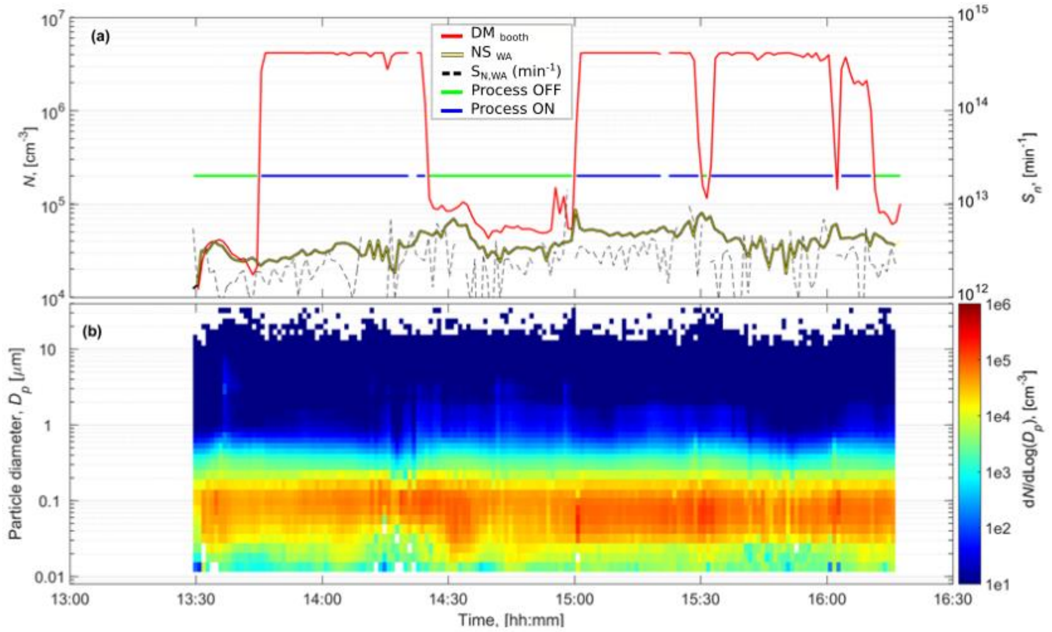

Figure S1. Booth #1, Day 1 a) shows particle number concentrations measured inside the booth by DiSCmini (DM), from worker area by NanoScan (WA) and particle emission rates solved by convolution from NanoScan WA concentrations. Blue line shows when the DM concentration was >10^5 cm^{-3} indicating that the plasma spray was ON and green line when the DM concentration was <10^5 cm^{-3} indicating that the plasma spray was OFF. Figure b) shows the particle size distributions measured by the NanoScan in the WA.

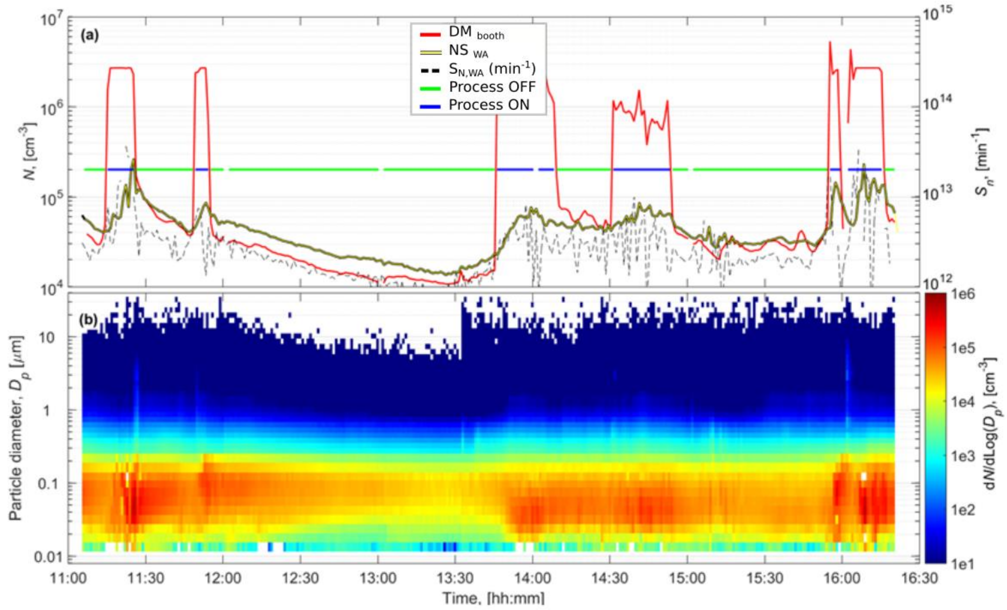

Figure S2. Booth #1, Day 2 a) shows particle number concentrations measured inside the booth by DiSCmini (DM), from worker area by NanoScan (WA) and particle emission rates solved by convolution from NanoScan WA concentrations. Blue line shows when the DM concentration was >105 cm-3 indicating that the plasma spray was ON and green line when the DM concentration was <105 cm-3 indicating that the plasma spray was OFF. Figure b) shows the particle size distributions measured by the NanoScan in the WA.

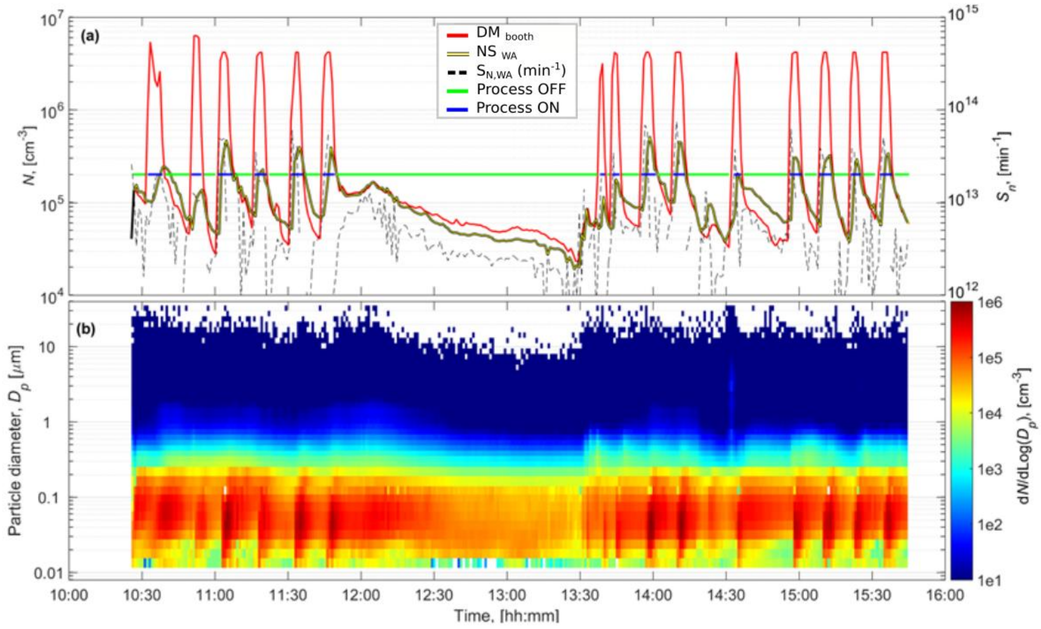

Figure S3. Booth #3, Day 4 a) shows particle number concentrations measured inside the booth by DiSCmini (DM), from worker area by NanoScan (WA) and particle emission rates solved by convolution from NanoScan WA concentrations. Blue line shows when the DM concentration was $>10^5$ cm^{-3} indicating that the plasma spray was ON and green line when the DM concentration was $<10^5$ cm^{-3} indicating that the plasma spray was OFF. Figure b) shows the particle size distributions measured by the NanoScan in the WA.

Table S1. Sampling day, booth, technic used (HVOF or APS), and feedstock materials summary. A: afternoon; M: morning. APS: atmospheric plasma spraying; HVOF: High Velocity Oxy-Fuel.

Day nº	Booth Model area	Spraying Technique	Shift	Feedstock Material	Composition
Day 1	#1	APS	A	Amdry 6228 & ANVAL 50/50	Al_2O_3 13TiO_2 & Cr/Ni
Day 2	#1	APS	M and A	Amdry 6228 & ANVAL 50/50	Al_2O_3 13TiO_2 & Cr/Ni
Day 3	#3	HVOF	M and A	WOKA 3702-1	WC 20Cr3C2 7Ni
Day 4	#3	HVOF	M and A	WOKA 3702-1	WC 20Cr3C2 7Ni

Table S2. Respirable mass concentration during the thermal spraying activity. Statistically significant increases are marked in bold.

Day	Period	Inside Booth	Worker Area (WA)	Inactivity (Background)
		Respirable ($\mu g\ m^{-3}$)	Respirable ($\mu g\ m^{-3}$)	Respirable ($\mu g\ m^{-3}$)
Booth #1 Model Area (Day 1)	Afternoon	n/a	**172**	53
Booth #1 Model Area (Day 2)	Morning<br>Afternoon	**130**<br>**169**	**161**<br>**123**	31
Booth #3 Model Area (Day 3)	Morning<br>Afternoon	**698**<br>**709**	**142**<br>**93**	26
Booth #3 Model Area (Day 4)	Morning<br>Afternoon	**522**<br>**367**	**171**<br>**136**	37

Table S3. Parameterization of the one-box model considering booth door open: V (m^3) is volume used for modeling, Q ($m^3\ h^{-1}$) is ventilation air volume flow through the Worker Area and ACH (h^{-1}) is the air changes per hour calculated from measured air speeds.

Model Area	*V*, [m^3]	*Q*, [m^3h^{-1}]	ACH, [h^{-1}]
#1	465	32155	69
#3	250	12291	49

Table S4. One Box modeled concentrations considering booth door open, and using the convolution theorem and the cyclic steady state (Cyclic SS) approach to calculate emission rate (S_N) from NanoScan data.

Day	Calculated S_N in WA (min^{-1})		Shift	Modeled concentrations (cm^{-3})		WA measured (cm^{-3})	Ratio (modeled/measured)	
	**Conv.**	**Cyclic SS**		**Conv.**	**Cyclic SS**		**Conv.**	**Cyclic SS**
Booth #1 Model Area (Day 1)	$1.4x10^{11}$	$1.3x10^{12}$	A	$1.4x10^{4}$	$1.6x10^{4}$	$4.2x10^{4}$	0.33	0.38
Booth #1 Model Area (Day 2)	$3.4x10^{12}$	$3.0x10^{12}$	M	$1.9x10^{4}$	$1.9x10^{4}$	$7.8x10^{4}$	0.24	0.24
			A	$2.1x10^{4}$	$2.0x10^{4}$	$4.9x10^{4}$	0.43	0.41
0.51B0.47o0.ot h #3 Model Area (Day 3)	$1.2x10^{13}$	$7.9x10^{12}$	M	$3.9x10^{4}$	$3.3x10^{4}$	$2.5x10^{5}$	0.16	0.13
			A	$4.0x10^{4}$	$3.3x10^{4}$	$9.0x10^{4}$	0.44	0.37
Booth #3 Model Area (Day 4)	$7.9x10^{12}$	$1.4x10^{13}$	M	$4.7x10^{4}$	$5.5x10^{4}$	$1.5x10^{5}$	0.31	0.37
			A	$4.5x10^{4}$	$5.1x10^{4}$	$1.3x10^{5}$	0.35	0.39
